# Editorial: The state of the art of person-centered healthcare: global perspectives

**DOI:** 10.3389/frhs.2025.1736241

**Published:** 2025-11-26

**Authors:** Vaibhav Tyagi, Tanya McCance, Claudia Rutherford, Brendan McCormack

**Affiliations:** 1 Susan Wakil School of Nursing and Midwifery, Faculty of Medicine and Health, The University of Sydney, Sydney, NSW, Australia; 2 School of Nursing and Paramedic Science, Ulster University, Belfast, United Kingdom

**Keywords:** person-centred healthcare, healthcare efficiency, pre-requisites, practice environment, macro context, person-centred processes, healthful cultures

**Figure 1:**
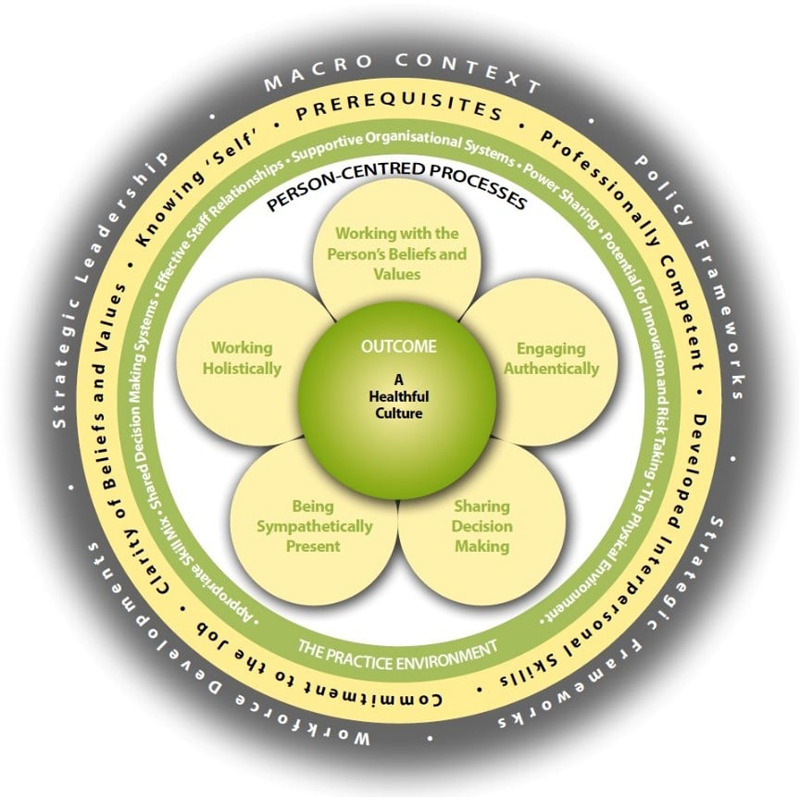
The Person-centred Practice Framework (Reproduced with permission from - McCormack and McCance).

Person-centred healthcare continues to gain momentum as a defining feature of quality care across the world. The global movement toward person-centredness transcends geographic and disciplinary boundaries. It calls for an authentic recognition of the personhood of all individuals engaged in health and care, whether as patients, families, professionals, or leaders. Despite decades of theoretical and empirical work, translating these principles into sustainable practice remains complex and challenging. This special issue, *The State of the Art of Person-Centred Healthcare: Global Perspectives*, brings together contemporary research from Europe, Australia, and beyond to present innovations, challenges, and innovations in advancing person-centred practice (PCP). The call for papers was framed around the Person-centred Practice Framework (PCPF, Figure 1), focusing on its five domains: prerequisites, practice environment, person-centred processes, outcomes, and macro-context. The twelve contributions in this issue collectively explore these dimensions through methodological diversity, ranging from meta-syntheses and scoping reviews to quantitative and mixed-methods research, practice development, and conceptual reflections. This collection of papers provides a comprehensive collection of new knowledge in person-centred practices, education, policy, leadership and measurement.

Several contributions demonstrate how organisational culture and leadership shape person-centredness. Teeling et al.’s paper on the Person-Centred Lean Six Sigma (PCLSS) model re-imagines quality improvement methodologies through a person-centred lens. Applied across multiple Irish healthcare settings, the model aligns operation with compassion, respect, and reflective practice – key characteristics of healthful cultures. Similarly, the work by Tuqiri et al. on *Co-Creating a Strategy for Transforming Person-Centred Cultures* showcases the power of facilitation and co-creation among nursing and midwifery leaders in designing a five-year roadmap for embedding person-centredness across a large local health district. Both exemplify how integrating person-centred principles into system-level improvement and workforce strategies can foster sustainable cultural transformation.

Complementing these system-level insights, the qualitative research by Vareta et al. (*Person-Centred Workplace Culture in an Inpatient Department for Older Adults with Chronic Illnesses*) reveals the tensions between routine-driven care and holistic approaches in day-to-day hospital practice. Their findings highlight how reflective dialogue and interprofessional collaboration remain essential in translating values into action. Together, these studies demonstrate that cultivating healthful cultures requires leadership at all levels and that creates links between operational structures, professional values, and the lived experience of patients and staff.

Leadership and education emerge as recurring catalysts for advancing person-centred practice. Haraldsdottir et al.’s *Developing Person-centred Care in Hospices through the Voice and Leadership of Nursing,* documents an emancipatory practice development program focusing on leadership in shaping person-centred care. Education's transformative potential is further supported by Tyagi et al. in *Implementation of Learning into Person-Centred Practice*, which presents quantitative evidence from community nursing programs. Their study found that integrating person-centred learning fosters key “prerequisites” of person-centred practice, especially clarity of beliefs and values, self-awareness, and interpersonal competence. Leadership also features prominently in Anker-Hansen et al.’s Mixed-Methods Systematic Review on Leadership Dynamics in Nursing Homes. Their synthesis underscores the pivotal role of leaders who model person-centred values, create shared visions, and distribute leadership to sustain engagement and care quality. Collectively, these contributions reaffirm that cultivating person-centred practice requires deliberate attention to leadership development, reflective learning, and the empowerment of practitioners as agents for change.

Moving from concepts and principles and their implementation, Forsgren et al. provide a comprehensive analysis of strategies and complexities underpinning PCP implementation. Their synthesis reveals the interplay between top-down policy imperatives and bottom-up co-creative processes, reiterating the need for flexible, iterative strategies that are context specific. Similarly, Mabire et al. offer an exemplary case of adapting person-centred frameworks to local contexts. Using concept mapping and implementation science, the study demonstrates how leadership support, participatory design, and ongoing training can facilitate the translation of theory into practice. These studies collectively position implementation not as a linear process but as a dynamic negotiation between values, evidence, and context.

As person-centred practice becomes a global policy aspiration, evaluating its impact remains a challenge. Rosted et al.’s paper on *Danish Translation and Cultural Adaptation of the PCPI-S and PCPI-C* addresses this by extending validated measurement tools into new linguistic and cultural settings. By offering reliable ways to assess both staff and patient perceptions of person-centredness, the study contributes to the growing international effort to build shared metrics for quality improvement and benchmarking. In the accompanying *Perspective Piece on Patient-Reported Outcomes in Evaluating Person-Centred Care*, Rutherford et al. argue for a re-examination of how person-centred outcomes are conceptualised and measured. They distinguish between patient- and person-reported outcomes, urging the field to capture what truly matters to individuals rather than what is easily quantifiable. Together, these contributions advance methodologies for evaluating person-centred cultures and practices.

Person-centredness is inherently relational and inclusive, yet its expression varies across cultures and systems. Son et al.’s Narrative Review on *Person-Centred Care for Migrants* illuminates the intersections of cultural sensitivity, migration, and person-centredness. Their review identifies three key practices - enhancing migrant participation, building intercultural partnerships, and promoting provider education, reinforcing the need for equity and cultural humility in person-centred care. Expanding the global perspective, Forsgren et al.’s Scoping Review on *Person-Centred Care as an Evolving Field of Research* offers a macro-level analysis of over 1,300 studies across six continents. They reveal the continuing ambiguity in terminology and the dominance of “patient-centred” discourse, which complicates synthesis and policy translation. This work calls for conceptual clarity and cross-disciplinary collaboration to strengthen the global coherence of the person-centred movement.

Taken together, the papers in this Research Topic illustrate both the maturity and the evolving challenges of person-centred healthcare. We identified three cross-cutting themes from this collection of work:

1. Integration across levels: Sustainable person-centred systems require alignment of values, leadership, education, and policy.
2. Measurement with meaning: Evaluation must move beyond checklists toward tools and metrics that capture human experience, context, and cultural diversity.
3. Co-creation and inclusivity: True person-centredness flourishes when all stakeholders (patients, professionals, and policymakers) are partners in shaping care.

As healthcare systems are shaped by increasing complexity, person-centredness provides conceptual, theoretical and practical frameworks for achieving excellence in healthcare. This collection of papers reflects the dynamic, collaborative, and interdisciplinary focus of global developments in person-centred healthcare. This collection of evidence demonstrates progress in this field and highlights the potential that exists in systematically advancing knowledge for the benefits of all persons.

